# Intersoftware variability in SPECT quality control: A technical note on analytical discrepancies and compliance decisions

**DOI:** 10.1002/acm2.70595

**Published:** 2026-04-29

**Authors:** Feng Jiawu, Zhang Li, Wang Shaojia, Liu Fei, Chen Yuhang, Peng Huaguang, Zhou Xuan, Cheng Yongliang, Sun Jingzhi

**Affiliations:** ^1^ Hubei Provincial Hospital of Integrated Chinese & Western Medicine (Hubei Provincial Hospital for Occupational Disease) Wuhan China; ^2^ Hubei Provincial Clinical Research Center for Pneumoconiosis and Poisoning Wuhan China; ^3^ Wuhan Prevention and Treatment Center for Occupational Diseases Wuhan China

**Keywords:** image analysis software, quality control, SPECT, standardization, variability

## Abstract

**Background:**

Quality control (QC) is essential for ensuring the diagnostic reliability of Single‐Photon Emission Computed Tomography (SPECT) systems. However, the reliance on third‐party software for analyzing QC metrics introduces a potential source of variability that is not yet standardized. Variability in QC results due to the use of different image analysis software may compromise both equipment evaluation and interinstitutional comparability.

**Purpose:**

This technical note assessed the variability in QC test results generated by different SPECT image analysis software packages to underscore the need for improved standardization.

**Methods:**

Five representative commercial SPECT QC software packages (A–E) were used to analyze identical DICOM image sets acquired from four SPECT/CT systems in accordance with the WS 523‐2019 standard. Evaluated metrics included file reading success rates, key performance indicators (intrinsic uniformity, resolution, linearity), and compliance rates. Statistical analysis employed ANOVA or Welch's tests, followed by LSD post hoc testing, with effect sizes (*η* ^2^) reported.

**Results:**

File reading success varied significantly (61.8%–100%), with Softwares B and D exhibiting higher failure rates. Compliance rates for identical devices varied considerably (68.8%–100%). Statistically significant intersoftware differences were found for intrinsic integral uniformity (*F* = 10.17, *p* < 0.05, *η* ^2 ^= 0.092), intrinsic spatial resolution (Welch *F* = 79.7, *p* < 0.05, *η* ^2 ^= 0.477), and intrinsic differential linearity (*F* = 2.65, *p* < 0.05, *η* ^2 ^= 0.137). The effect sizes for spatial resolution and differential linearity indicated large effects (*η* ^2^ > 0.14). Significant differences (*p* < 0.05) in key indicators were also observed across analyses for UFOV/CFOV fields of view and *X*/*Y* directions. Pairwise comparisons indicated that the primary differences existed between Softwares B, D, and E compared to the other packages.

**Conclusion:**

We found significant disparities between software packages in both file reading capability and the analysis of key QC performance indicators. These differences directly impact the accuracy of equipment performance evaluations and interinstitutional comparability, potentially leading to divergent conclusions regarding the same device's compliance status.

## 1 INTRODUCTION

Rigorous quality control (QC) is essential to ensure the quantitative accuracy and diagnostic reliability of Single‐Photon Emission Computed Tomography (SPECT). This process is guided by internationally recognized standards such as those from the National Electrical Manufacturers Association (NEMA) NU series.^1^, ^2^ In 2019, the Chinese National Health Commission issued the industry standard WS 523‐2019, “Specifications for Quality Control Testing of Gamma Cameras and Single‐Photon Emission Computed Tomography Systems,”^3^ which is largely adapted from NEMA NU principles. While standards like WS 523‐2019 define testing procedures and general data processing principles, the critical computation and analysis of core performance metrics—such as intrinsic uniformity, spatial resolution, and linearity—are typically performed by third‐party image analysis software.

Software heterogeneity is a recognized source of variability in QC results.^4^, ^5^ Differences can arise in DICOM parsing, field‐of‐view (FOV) definition, region‐of‐interest (ROI) placement, preprocessing, and the implementation of core algorithms (e.g., for uniformity calculations, FWHM fitting, and LSF compensation).^6^, ^7^, ^8^, ^9^, ^10^ These uncontrolled variations can bias performance evaluations and compromise interinstitutional comparability, thereby hindering regional QC assessments and effective oversight.^11^, ^12^ Although studies such as that by Edam et al.^13^ have identified factors like pixel size discrepancies affecting uniformity, a comprehensive evaluation of mainstream commercial software under a unified national standard is lacking.

This technical note addresses this gap by the following: (1) evaluating multiple critical performance metrics; (2) focusing on mainstream commercial software used in technical service markets; (3) employing rigorous statistics to quantify variability; and (4) using the WS 523‐2019 protocol. We assess software across the following three dimensions: file compatibility, analytical consistency, and compliance decision agreement. By quantifying this intersoftware variability, our findings aim to inform urgent standardization efforts for SPECT QC software algorithms.

## 2 Methods

### 2.1 Software

This study selected five radiological health technical service institutions certified for SPECT QC testing and their respective commercial SPECT QC image analysis software as research subjects. The software packages were as follows: Software A (SPECT V6, version i Lab V6.1; Beijing Zean Technology Co., Ltd.), Software B (SPECT‐QC Image Quality Software, version V3.0; Beijing Cardinor Technology Co., Ltd.), Software C (SPECT Quality Control Software, version PLK‐HW001; Beijing Pulinkang Technology Co., Ltd.), Software D (SPECT Image Analysis Software, version 1.0; Hubei Zhongzhi Technology Co., Ltd.), and Software E (SPECT Image Analysis Software, version 2.0; Beijing Kangweiruide Co., Ltd.). All five software packages were the latest official release versions provided by the manufacturers at the time of the study, and all are claimed by their vendors to comply with the WS 523–2019/NEMA standards. Detailed information on the vendor, official website, technical basis, and regulatory status of all included software packages is provided in Supplementary Material 1: Detailed Information on SPECT QC Software Packages.

### 2.2 Image data

QC images were acquired from four dual‐head SPECT/CT systems (GE Discovery NM/CT670, 670 Pro; Siemens Symbia Intevo Bold, Intevo 6) using standard test phantoms that comply with the requirements of WS 523‐2019 and NEMA NU1‐2023. The phantoms (model SRT‐140) included the following: a lead mask with 1‐mm‐wide parallel slits (one with slits in the *X*‐direction and one in the *Y*‐direction), a 150‐mm‐diameter flat plastic dish, a dual‐line source phantom consisting of two capillary tubes (each with an inside diameter of 1 mm and an active filled length of at least 120 mm), and a flood source phantom. All acquisitions strictly followed the WS 523‐2019 protocol.

The radiotracer used for all QC acquisitions was ^99^Tc^m^‐pertechnetate. Imaging protocols were standardized as follows: a 20% energy window centered at 140 keV; static images; tomographic images acquired with a 360° rotation (6° per step); and whole‐body images acquired with a scan speed of 15 cm/min. The analysis dataset consisted of original DICOM files from static, tomographic, and whole‐body acquisitions.

### 2.3 Performance metrics

Metrics analyzed per WS 523‐2019 included: intrinsic integral/differential uniformity (%), intrinsic spatial resolution (FWHM, mm), intrinsic differential/absolute linearity (mm), system spatial resolution (FWHM, mm), tomographic spatial resolution (FWHM, mm), and whole‐body spatial resolution (FWHM, mm), assessed over UFOV/CFOV and *X*/*Y*‐directions.

### 2.4 Statistical analysis

Analyses used SPSS 26.0. Continuous data are mean ± SD. Intersoftware differences were assessed using the Levene's test for variance homogeneity, followed by one‐way ANOVA (for homogeneous variances) or Welch's ANOVA (for heterogeneous variances). The *F*‐statistic, which quantifies the ratio of between‐group variance to within‐group variance, was used to test for significant differences in metric means across the software packages (significance threshold: *p* < 0.05). Significant *F*‐tests were followed by LSD post hoc tests with Bonferroni correction. Effect size was quantified using *η* ^2^ (small ≥ 0.01, medium ≥ 0.06, large ≥ 0.14)^14^. Compliance rates against WS 523‐2019 limits were calculated for each software package.

## 3 RESULTS

### 3.1 General findings

The file‐reading success rates varied significantly across the five software packages (Table 1). Softwares A, C, and E successfully processed 100% of files. In contrast, Software B (84.6%–91.7%) and Software D (56.3%–75.0%) exhibited notable failure rates, particularly with high‐count‐rate images, which precluded subsequent sensitivity calculations.

**TABLE 1:** Success rates of metric extraction from QC image files by analysis software.

Sample ID	No. files	Total metrics	Software A	Software B	Software C	Software D	Software E
Sample 1	15	55	55 (100%)	47 (85.5%)	55 (100%)	34 (61.8%)	55 (100%)
Sample 2	15	55	55 (100%)	47 (85.5%)	55 (100%)	33 (60.0%)	55 (100%)
Sample 3	11	48	48 (100%)	44 (91.7%)	48 (100%)	27 (56.3%)	48 (100%)
Sample 4	14	52	52 (100%)	44 (84.6%)	52 (100%)	39 (75.0%)	52 (100%)

*Note*: The number of files (Table 1) represents the total DICOM objects acquired.

Compliance rates for identical devices and metrics also varied substantially between software packages (Table 2). The ranges were as follows: Softwares A (84.4%–100%), B (71.9%–100%), C (87.5%–100%), D (68.8%–100%), and E (78.1%–100%). Marked discrepancies were observed for intrinsic integral uniformity (87.5%–100%), intrinsic spatial resolution (where Software D had a 68.8% compliance rate vs. 100% for others), and intrinsic linearity metrics.

**TABLE 2:** Compliance rates (%) by QC metric and software.

Metric						
Intrinsic uniformity	*n*	Software A	Software B	Software C	Software D	Software E
– Integral uniformity	16	87.5%	100.0%	93.8%	93.8%	93.8%
– Differential uniformity	16	87.5%	93.8%	87.5%	87.5%	87.5%
Intrinsic spatial resolution	32	100.0%	100.0%	100.0%	68.8%	100.0%
Intrinsic linearity						
– Differential linearity	32	84.4%	71.9%	87.5%	75.0%	78.1%
– Absolute linearity	32	96.9%	93.8%	100.0%	68.8%	90.6%
Whole‐body spatial resolution	12	100.0%	100.0%	100.0%	100.0%	100.0%
Tomographic spatial resolution	12	100.0%	100.0%	100.0%	100.0%	100.0%

*Note*: The number of metrics (*n*) in Table 2 refers to the number of analyzable results successfully extracted from these files.

### 3.2 Variability in key performance indicator analyses

Significant intersoftware differences were found for critical intrinsic metrics (Table 3).

**TABLE 3:** Comparative analysis of QC metrics across fields of view.

Metric	Software A	Software B	Software C	Software D	Software E	*F*	*p*	*η* ^2^
Integral uniformity (%)	3.38 ± 0.99	1.70 ± 0.64^a^	3.27 ± 0.96	3.21 ± 0.90	3.25 ± 0.91	10.17	0.000	0.092
UFOV	3.16 ± 0.98	1.48 ± 0.57^a^	3.06 ± 1.00	2.93 ± 0.82	3.12 ± 0.99	5.184	0.002	0.364
CFOV	3.60 ± 1.01	1.92 ± 0.67^a^	3.48 ± 0.94	3.48 ± 0.94	3.38 ± 0.87	5.003	0.003	0.372
Differential uniformity (%)	2.15 ± 1.18	1.90 ± 0.96	2.10 ± 1.20	1.90 ± 0.87	2.08 ± 1.21	0.184	0.946	–
UFOV	1.84 ± 0.50	1.91 ± 1.00	2.14 ± 1.23	1.99 ± 1.02	2.10 ± 1.24	0.199	0.975	–
CFOV	2.08 ± 1.25	1.91 ± 1.00	2.07 ± 1.26	1.80 ± 0.77	2.06 ± 1.26	0.097	0.983	–
Intrinsic spatial resolution (mm)	3.73 ± 0.07	3.64 ± 0.05	3.67 ± 0.05	2.95 ± 0.88^a^	4.09 ± 0.09^a^	79.7^b^	0.000	0.477
UFOV	3.77 ± 0.06	3.70 ± 0.04	3.70 ± 0.04	2.98 ± 0.92^a^	4.11 ± 0.08	42.68^b^	0.000	0.470
CFOV	3.69 ± 0.05	3.62 ± 0.05	3.64 ± 0.04	2.91 ± 0.91^a^	4.06 ± 0.10	33.94^b^	0.000	0.484
Intrinsic differential linearity (mm)	0.14 ± 0.07	0.17 ± 0.08	0.13 ± 0.07	0.11 ± 0.06^a^	0.19 ± 0.05^a^	2.653	0.040	0.137
UFOV	0.16 ± 0.08	0.20 ± 0.08	0.14 ± 0.07	0.13 ± 0.08	0.20 ± 0.06	1.447	0.242	–
CFOV	0.12 ± 0.07	0.15 ± 0.08	0.12 ± 0.07	0.09 ± 0.04	0.17 ± 0.04	1.333	0.280	–
Intrinsic absolute linearity (mm)	0.31 ± 0.15	0.34 ± 0.14	0.23 ± 0.16	0.37 ± 0.24	0.36 ± 0.13	1.703	0.600	–
UFOV	0.38 ± 0.17	0.38 ± 0.15	0.31 ± 0.18	0.48 ± 0.30	0.41 ± 0.13	0.66	0.624	–
CFOV	0.24 ± 0.09	0.29 ± 0.11	0.16 ± 0.09	0.27 ± 0.11	0.31 ± 0.12	2.599	0.055	–

^a^ Significant difference identified by LSD post hoc test (*p* < 0.05)

^b^ Welch ANOVA used when variances were unequal.

Intrinsic integral uniformity showed significant variation (*F* = 10.17, *p* < 0.05, *η* ^2^ = 0.092). Software B reported systematically lower values (1.70% ± 0.64%) than others (3.21%–3.38%; *p* < 0.05), consistent across UFOV/CFOV.

Intrinsic spatial resolution exhibited the largest differences (Welch *F* = 79.7, *p* < 0.05; *η* ^2^ = 0.477). Software D yielded lower values (2.95 ± 0.88 mm) with high dispersion, while Software E yielded higher values (4.09 ± 0.09 mm) than A, B, C (*p* < 0.05).

Intrinsic differential linearity varied significantly (*F *= 2.65, *p* < 0.05, *η* ^2^ = 0.137). Software D reported lower values (0.11 ± 0.06 mm) and Software E higher values (0.19 ± 0.05 mm) compared to others.

No significant differences (*p* > 0.05) were found for system spatial resolution, whole‐body spatial resolution, tomographic spatial resolution, intrinsic differential uniformity, or intrinsic absolute linearity.

Analysis by spatial direction (Table 4) confirmed significant directional variability for intrinsic spatial resolution (*X*/*Y*) and differential linearity (*X*). Disparities primarily originated between Software B/D/E and others (A/C).

**TABLE 4:** Comparative analysis of QC metrics across spatial directions (*X*/*Y*).

Metric	Software A	Software B	Software C	Software D	Software E	*F*	*p*	*η* ^2^
System spatial resolution								
*X*	7.33 ± 0.55	7.19 ± 0.49	7.24 ± 0.54	7.03 ± 0.46	6.78 ± 0.46	0.714	0.597	–
*Y*	7.66 ± 0.84	7.53 ± 0.85	7.57 ± 0.86	7.17 ± 0.67	6.98 ± 0.79	0.463	0.761	–
Whole‐body spatial resolution								
*X*	8.30 ± 1.37	8.25 ± 1.30	8.32 ± 1.30	7.24 ± 0.99	8.80 ± 0.86	0.843	0.513	–
*Y*	9.00 ± 1.37	8.88 ± 1.48	8.98 ± 1.54	8.90 ± 1.73	9.30 ± 1.14	0.073	0.990	–
Tomographic spatial resolution								
Transverse	12.2 ± 2.58	12.0 ± 2.09	12.3 ± 2.14	12.0 ± 2.12	12.5 ± 2.37	0.053	0.994	–
Axial	13.0 ± 2.10	12.4 ± 1.96	12.3 ± 2.14	12.6 ± 1.67	12.4 ± 1.99	0.088	0.985	–
Intrinsic spatial resolution								
*X*	3.76 ± 0.09	3.65 ± 0.07^a^	3.69 ± 0.08	4.08 ± 1.06^a^	4.10 ± 0.10^a^	36.66^b^	0.000	0.150
*Y*	3.70 ± 0.05^a^	3.63 ± 0.05^a^	3.64 ± 0.03	3.64 ± 0.06	4.07 ± 0.08^a^	28.03^b^	0.000	0.489
Intrinsic differential linearity								
*X*	0.14 ± 0.08	0.19 ± 0.10	0.13 ± 0.08	0.07 ± 0.05^a^	0.18 ± 0.05	37.04^b^	0.000	0.257
*Y*	0.14 ± 0.07	0.16 ± 0.07	0.14 ± 0.07	0.12 ± 0.06	0.19 ± 0.06^a^	2.50	0.051	0.130
Intrinsic absolute linearity								
*X*	0.31 ± 0.15	0.39 ± 0.18^a^	0.21 ± 0.19^a^	0.27 ± 0.20	0.29 ± 0.10	2.55	0.046	0.120
*Y*	0.31 ± 0.19	0.28 ± 0.12	0.26 ± 0.14^a^	0.42 ± 0.23	0.43 ± 0.24	2.57	0.046	0.133

^a^ Significant difference identified by LSD post hoc test (*p* < 0.05).

^b^ Welch ANOVA used when variances were unequal;.

### 3.3 Intersoftware consistency and bias analysis

**FIGURE 1:**
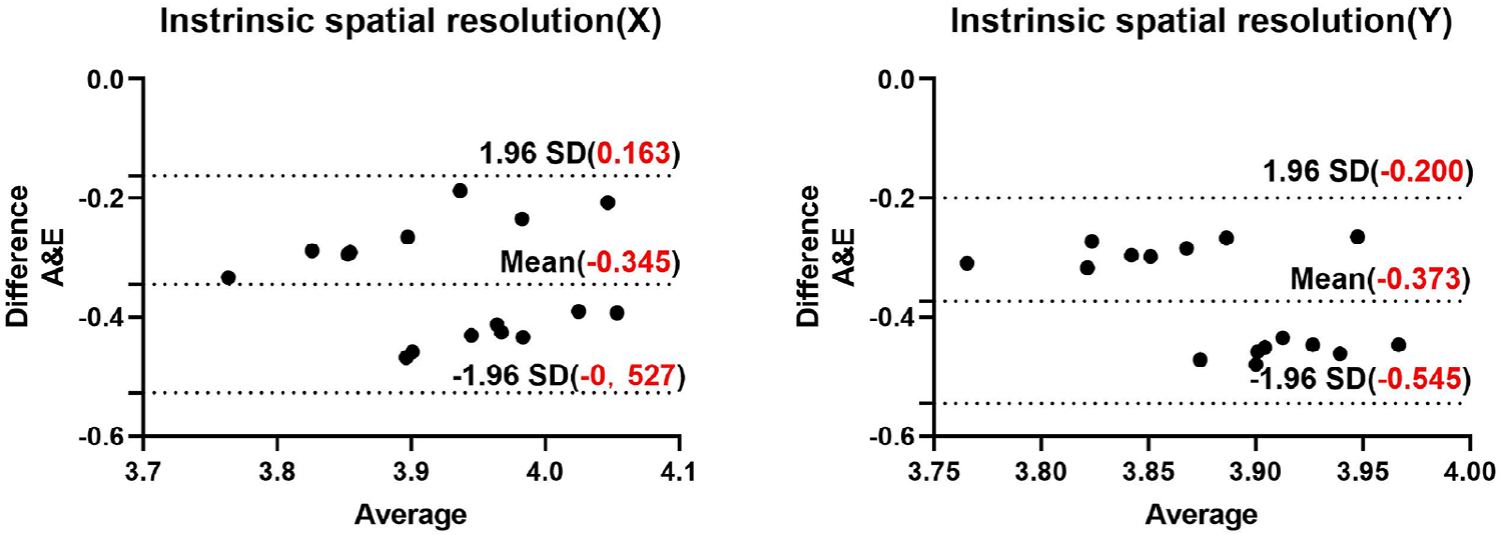
Bland–Altman plots for intrinsic spatial resolution. (a) *X*‐direction, Software A versus E; (b) *Y*‐direction, Software A versus E.

Bland–Altman analyses for metrics with significant differences confirmed systematic biases:

Intrinsic spatial resolution (Figure 1a,b): Software E showed positive bias versus Software A (mean: −0.345 mm [*X*], −0.373 mm [*Y*]), risking misclassification (e.g., incorrectly flagging compliant devices as noncompliant, or vice versa).

**FIGURE 2:**
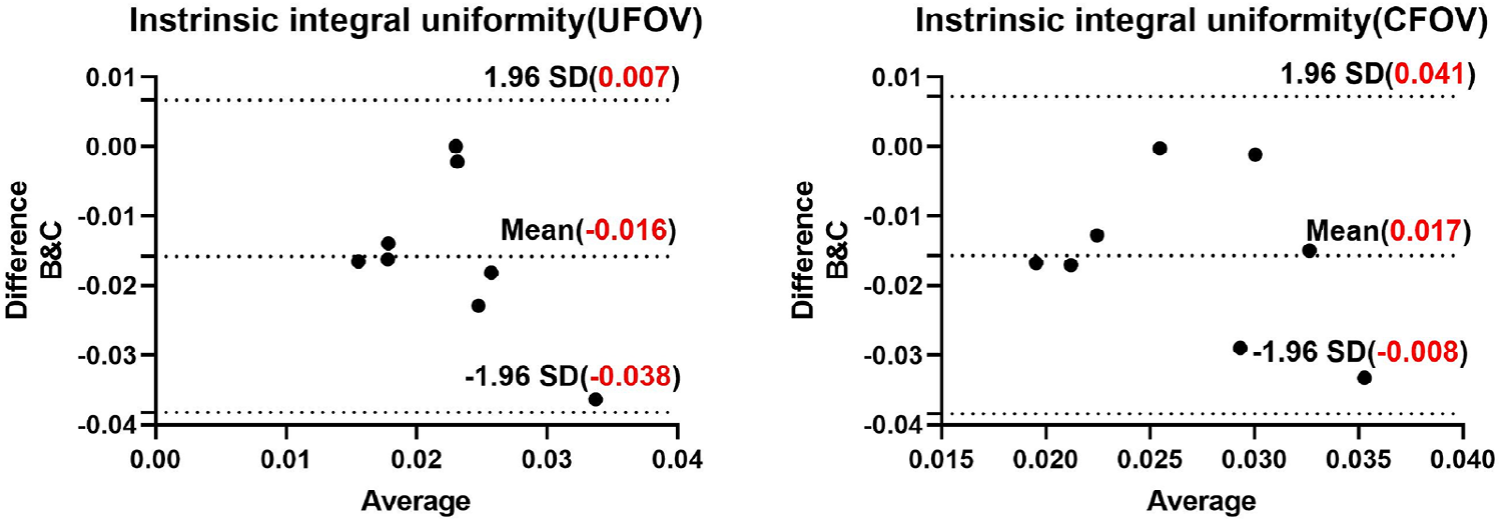
Bland–Altman plots for intrinsic integral uniformity. (a) UFOV, Software B versus C; (b) CFOV, Software B versus C.

Intrinsic integral uniformity (Figure 2a,b): Software B exhibited systematically lower values relative to Software C (mean bias: −1.68% for both UFOV and CFOV). This confirms a consistent underestimation bias in Software B's calculation of intrinsic integral uniformity.

**FIGURE 3:**
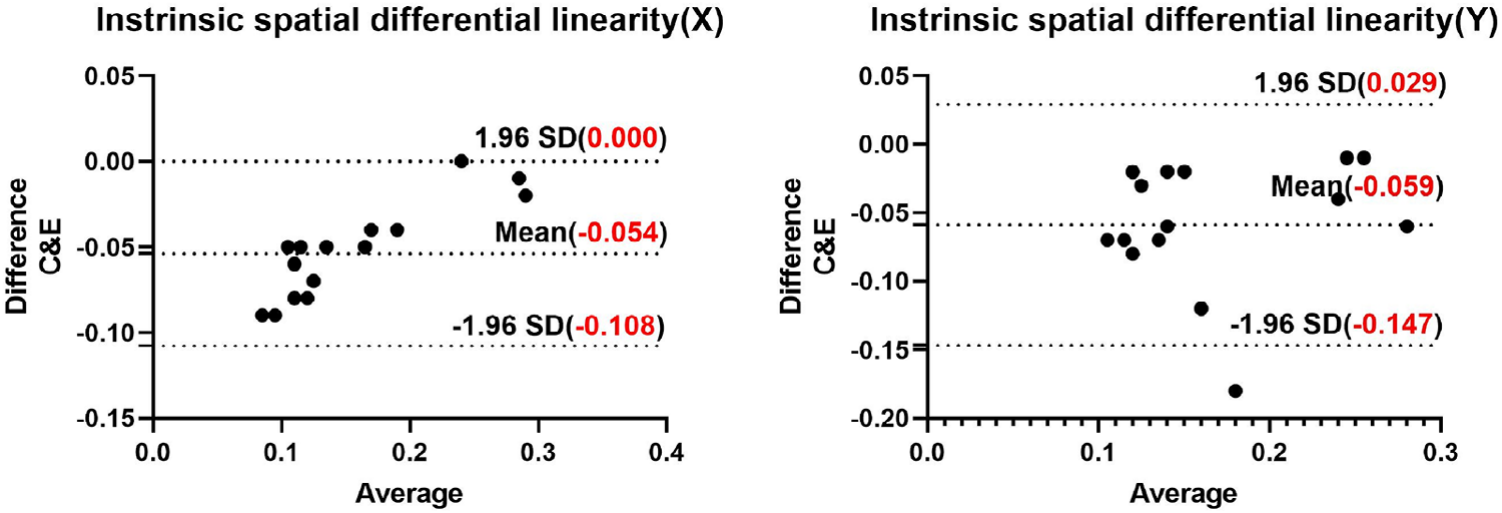
Bland–Altman plots for intrinsic differential linearity. (a) *X*‐direction, Software C versus E; (b) *Y*‐direction, Software C versus E.

Intrinsic differential linearity (Figure 3a,b): Software E showed systematically higher values compared to Software C (mean bias: +0.054 mm [*X*‐direction]; +0.059 mm [*Y*‐direction]). Notably, the analytical deviations for some samples approached the acceptance thresholds defined by the standard.

## 4 DISCUSSION

This technical note demonstrates significant intersoftware variability in SPECT QC, affecting file reading, analytical results, and compliance decisions. Consequently, different software packages can yield divergent pass/fail verdicts for the same device, which has direct implications for equipment management and patient safety.

The observed systematic variations likely stem from algorithmic divergences in the following four critical domains:

1. FOV definition and processing: Inconsistencies in UFOV/CFOV auto‐identification algorithms, nonuniformity correction regions, ROI size/shape/placement protocols, and directional (*X*/*Y*) ROI segmentation logic.^2^

2. Image preprocessing workflows: Heterogeneity in smoothing filters, background subtraction strategies, noise filtering algorithms, and pixel saturation handling—particularly for high‐count‐rate images—potentially explains the read failures observed in Softwares B and D.^15^, ^16^, ^17^ The reasons for file reading failures, particularly for Software B (SPECT‐QC) and Software D (SPECT Image Analysis Software), remain unclear without access to their proprietary code. Future collaboration with vendors is necessary to diagnose these specific incompatibilities (e.g., handling of proprietary DICOM tags or high‐count‐rate saturation).

3. Core algorithm implementation: (a) Uniformity metrics: Variations in the formulae used to calculate integral and differential uniformity, including pixel extremum screening rules (e.g., exclusion thresholds), normalization methods, and statistical windowing approaches.^18^, ^19^ (b) Spatial resolution (FWHM): Discrepancies in methodologies for fitting line/point spread functions (LSFs/PSFs) (e.g., Gaussian vs. iterative approaches), peak localization algorithms, FWHM computation, and edge‐effect correction strategies^20^, ^21^—likely driving the extreme deviations observed in Softwares D and E. (c) Linearity: Differences in techniques for LSF extraction, distortion compensation algorithms, and principles for peak identification, potentially explaining the elevated differential linearity values reported by Software E.^22^ While these factors represent probable sources, the proprietary nature of commercial software obscures definitive attribution of the observed differences to specific algorithmic components (e.g., ROI segmentation logic, fitting specifics)—necessitating vendor collaboration for transparent validation.

4. Phantom/acquisition adaptability: Despite standardized acquisitions, subtle differences in phantom conditions or vendor‐specific protocols (e.g., GE vs. Siemens systems, tomographic reconstruction parameters) may expose software‐specific vulnerabilities due to suboptimal algorithm calibration, amplifying intersoftware differences.^23^

The observed variability was predominantly associated with intrinsic metrics (uniformity, spatial resolution, linearity) rather than system or tomographic metrics. This discrepancy can be attributed to the differing physical and algorithmic contexts of these tests. Intrinsic metrics are derived directly from the uncollimated detector's response, making them highly sensitive to software‐specific implementations of complex calculations, such as global extremum searching for uniformity or LSFs fitting for resolution. In contrast, system spatial resolution is overwhelmingly governed by the collimator, a physical component whose dominant effect likely masks the subtler variations introduced by software analysis. Similarly, for tomographic metrics, the reconstruction algorithms and filters (e.g., OSEM, FBP) may have a homogenizing effect that overshadows pre‐reconstruction analytical differences between software packages. The robustness of intrinsic differential uniformity may stem from its more localized and potentially standardized calculation over a smaller pixel matrix, reducing the impact of algorithmic choices. While these considerations provide a plausible framework for interpreting our results, the proprietary nature of the software algorithms precludes definitive conclusions. Future work with access to underlying code is needed to confirm these hypotheses.

The substantial fluctuations in compliance rates for intrinsic uniformity and linearity metrics observed in this study (68.8%–100%) closely align with reports from other regions: Asian data indicating intrinsic uniformity/linearity pass rates as low as 38.5%,^24^ and Australian studies documenting significant interinstitutional variability in SPECT QC testing.^25^, ^26^ Notably, the “sensitivity to count conditions” hypothesis proposed by Yu et al.^27^ is directly substantiated by the failure of Softwares B and D to process high‐count‐rate images in our study.

Our findings significantly extend the work of Edam et al. (2018),^13^ who identified pixel size as a factor in uniformity analysis. We demonstrate that commercial software variability is more profound, involving core algorithmic differences in spatial resolution (FWHM fitting) and linearity assessment, with large effect sizes (*η* ^2^ > 0.14). Unlike the freeware in their study, which agreed with baseline calculations, the commercial software we tested showed systematic biases (e.g., Software B's underestimation, Software E's overestimation) that directly threaten pass/fail decision accuracy. This highlights that the “black box” nature of commercial algorithms, beyond configurable parameters, is a major obstacle to QC standardization.

These discrepancies lead to risk misclassification, potentially resulting in unnecessary servicing of compliant systems or the continued use of substandard equipment. This wastes resources and compromises patient care. To address these issues, we propose the following recommendations:

1. Enhanced standards: Standardization bodies should incorporate detailed technical specifications for core metric algorithms (formulae, ROI rules, preprocessing, fitting methods) into SPECT QC standards. Developing open‐source reference algorithm libraries is crucial for benchmarking.^28^

2. Shared DICOM QC image repositories—spanning multivendor equipment, diverse phantom states, and varied acquisition protocols, including edge‐case scenarios like high count rates—must be established nationally and internationally. These repositories would enable: consistency validation, software certification, and ongoing quality assurance.^29^, ^30^

3. Recommendations for software developers:

a. Adherence and validation: Actively implement refined international/national standards in software development and updates. Conduct rigorous algorithm verification and consistency testing against: (i) standard reference tools (e.g., MATLAB/Python reference implementations), (ii) gold‐standard methodologies. Publicly disclosing comparative results and deviation reports would enhance credibility.

b. Technical optimization: Continuously improve DICOM parsing capabilities to ensure robust compatibility with high‐count‐rate images and multivendor equipment outputs.^31^ Integrate automated anomaly detection with alerts for ROI misplacement, fitting failures, and count saturation events.

4. End‐user implementation guidelines (technical service institutions and hospitals):

a. Software selection criteria: Prioritize QC software validated through independent verification by accredited bodies (e.g., AAPM, EANM) or compliance with rigorous international standards (e.g., AAPM TG267). Require vendors to provide detailed algorithm compliance validation reports.^32^

b. Reporting transparency: Mandate explicit documentation in QC reports of the software name, version, and critical configurable parameters.

These discrepancies are not merely academic; they translate directly to clinical practice. For instance, a systematic overestimation of spatial resolution (as seen with Software E) could lead to the acceptance of a system whose degraded resolution might blur small lesions, potentially affecting diagnostic sensitivity for diseases like Parkinson's or coronary artery disease. Conversely, underestimation of uniformity (Software B) might trigger unnecessary service calls on a properly functioning camera, wasting valuable clinical time and resources.

Study limitations include the following: (1) commercial software coverage excluded key platforms (e.g., Hermes, Segami); (2) the impact of software version upgrades was not tested; (3) potential interactions between reconstruction algorithms (e.g., OSEM vs. FBP) and QC metric results were not assessed. It is important to note that this study was designed to quantify intersoftware variability, not to determine absolute accuracy. In the absence of a universally accepted reference algorithm or a certified physical phantom with known metric values, we did not identify any software as the “ground truth.” The significant differences observed, regardless of which software might be more accurate, underscore the need for algorithm standardization. Future work using digital reference objects or simulated images with precisely known parameters could help establish benchmarks for accuracy.

## 5 CONCLUSION

Current SPECT QC software exhibits substantial disparities in file compatibility, analytical results, and compliance decisions for identical devices. Key metrics, including intrinsic uniformity, spatial resolution, and linearity, showed statistically significant variations. These discrepancies directly impact equipment pass/fail decisions and longitudinal performance monitoring. To enhance reliability and comparability, we urgently recommend the standardization of algorithm specifications within QC protocols, the development of open‐source reference libraries, and the mandatory validation of software against benchmark datasets.

## AUTHOR CONTRIBUTIONS

Feng Jiawu and Zhang Li are co‐first authors of this article. Feng Jiawu, Zhang Li, and Sun Jingzhi contributed to the design of the study and discussed the data. Liu Fei, Peng Huaguang, and Chen Yuhang planned, performed, and analyzed the measurements. Feng Jiawu, Zhang Li, Wang Shaojia, and Zhou Xuan were the major contributors in writing the manuscript. All authors discussed the results and implications and commented on the manuscript. All authors read and approved the final manuscript.

## CONFLICT OF INTEREST STATEMENT

The authors declare no conflicts of interest. The study was conducted impartially. The selection of the software packages for evaluation was based solely on their prevalence in the regional technical service market and their claim of compliance with the WS 523–2019 standard. No software vendor or manufacturer was involved in the study design, data analysis, interpretation, or writing of the manuscript.

## ETHICS STATEMENT

Not applicable (phantom study only, no human or animal subjects involved).

## Data Availability

Authors will share data upon reasonable request to the corresponding author. The detailed software information is provided in Supplementary Material 1 attached to this manuscript.
